# Antioxidant Defenses: A Context-Specific Vulnerability of Cancer Cells

**DOI:** 10.3390/cancers11081208

**Published:** 2019-08-20

**Authors:** Jordan A. Cockfield, Zachary T. Schafer

**Affiliations:** Department of Biological Sciences, University of Notre Dame, Notre Dame, IN 46556, USA

**Keywords:** antioxidants, reactive oxygen species, cancer, metabolism, Nrf2, ECM-detachment, antioxidant enzymes

## Abstract

Reactive oxygen species (ROS) are well known for their capacity to cause DNA damage, augment mutagenesis, and thereby promote oncogenic transformation. Similarly, agents that reduce ROS levels (antioxidants) are frequently thought to have anti-cancer properties given their propensity to minimize DNA damage and mutagenesis. However, numerous clinical studies focused on antioxidants suggest that this is a facile premise and that antioxidant capacity can be important for cancer cells in a similar fashion to normal cells. As a consequence of this realization, numerous laboratories have been motivated to investigate the biological underpinnings explaining how and when antioxidant activity can potentially be beneficial to cancer cells. Relatedly, it has become clear that the reliance of cancer cells on antioxidant activity in certain contexts represents a potential vulnerability that could be exploited for therapeutic gain. Here, we review some of the recent, exciting findings documenting how cancer cells utilized antioxidant activity and under what circumstances this activity could represent an opportunity for selective elimination of cancer cells.

## 1. Introduction

Dietary intake of compounds such as vitamin A, vitamin C, vitamin E and β-carotene has long been associated with a diminished risk of developing a variety of human cancers [1,2,3]. It has been postulated that the primary mechanism of anti-tumor activity by these compounds is their capacity to function as antioxidants; which, by definition, function to eliminate reactive oxygen species (ROS) that can damage DNA and lead to mutagenesis. There are a number of biological factors that can contribute to cancer cells becoming oxidatively stressed. These include (but are not limited to): dysfunctional or abnormal oxidative phosphorylation in the mitochondria, deficient generation of reducing equivalents during extracellular matrix (ECM)-detachment, and infiltration of ROS-producing inflammatory cells [4,5]. The physiological role of ROS in these vastly distinct contexts has been the subject of intense study for a number of years. For example, in some cases, ROS can function to activate signal transduction pathways that activate gene expression, increase cell proliferation, or promote survival [6]. However, in other cases, elevated levels of ROS can cause DNA damage and the induction of apoptosis [7]. Nonetheless, the prevailing dogma that neutralizing ROS can prevent the accumulation of oncogenic mutations that contribute to tumorigenesis has long guided the view of the general public concerning dietary intake of compounds with antioxidant capacity [5].

Curiously, clinical studies examining the hypothesis that dietary intake of antioxidants antagonizes or limits tumorigenesis suggest that this premise is overly simplistic in some cases and outright incorrect in others. For instance, in 1985, an eight-year clinical trial including over twenty-nine thousand male smokers between the ages of 50 to 69 was executed to investigate the anti-cancer effects of beta carotene (BC), a precursor of vitamin A with strong antioxidant properties [8,9]. After being randomly assigned to receive 20 mg/day of BC (three times the recommended dietary allowance, RDA) the result of this clinical trial was an 18% percent increase in the incidence of lung cancer in male smokers who consumed BC. Similarly, in 1996, over eighteen thousand smokers, former smokers, and workers previously exposed to asbestos were involved in a randomized, double-blind, placebo-controlled primary prevention trial focused on investigating the efficacy of BC/vitamin A supplementation on lung cancer incidence [10]. Participants randomly selected to receive the active supplements consumed 30 mg of BC and 25,000 IU (15 mg) of vitamin A daily. Due to an increase in the risk of both lung cancer diagnosis and mortality, the trial was discontinued 21 months prior to the expected end date. Moreover, the report of the Selenium and Vitamin E Cancer Prevention Trial (SELECT), conducted between 2004 and 2011, found that the risk of prostate cancer for healthy men was significantly increased upon administration of 400 IU/day of vitamin E supplements [11]. Taken together, the results from these clinical trials suggest that dietary antioxidants may, at times, contribute to (rather than impede) tumor progression.

The fact that the data obtained from these clinical trials do not lead to a general conclusion regarding links to the consumption of dietary antioxidants and tumor development is perhaps not surprising. Indeed, understanding the basic biological mechanisms that may underlie these findings is complicated by a number of factors. The selection of specific antioxidant molecules, the limited number of agents tested to date, and the use of pharmacological (in lieu of dietary) doses all complicate the interpretation of these data and any efforts to make more generalized conclusions [12]. That being said, these interesting clinical trials have motivated a number of laboratories to investigate the biological underpinnings explaining how and when antioxidant compounds can promote tumorigenesis. Given that mammalian cells (both normal and cancerous) are endowed with sophisticated antioxidant defense systems to mitigate ROS levels, many of these studies focus on understanding both intrinsic and extrinsic mechanisms detailing how cancer cells regulate ROS levels under certain stressful conditions. While the context varies significantly, there has been significant in vitro and in vivo data that lead to a unifying conclusion: antioxidant activity can, in certain cases, abet cancer cells in a similar fashion to non-cancerous cells. This supposition may provide a mechanistic rationale for the interesting findings in the aforementioned clinical trials. More importantly, the reliance of cancer cells on intrinsic and extrinsic mechanisms that modulate antioxidant activity suggests that targeting antioxidant pathways may be a vulnerability that could provide efficacious when considering treatment regimens for cancer patients. In this review, we focus on the accumulating evidence suggesting that cancer cells rely on antioxidant activity to navigate stressful environments during tumor progression.

## 2. Antioxidant Activity, Chemotherapy, and Tumor Progression

### 2.1. Antioxidants, Nrf2 Signaling, and Chemotherapeutic Resistance

For many years, numerous laboratories have investigated the possibility that antioxidant compounds, when delivered in combination with certain cancer therapies, could function synergistically to eliminate cancer cells. There have certainly been promising findings amid these efforts. For example, a study utilizing the antioxidants pyrrolidinedithiocarbamate (PDTC) and vitamin E in combination with DNA-damaging chemotherapeutic agents (5-fluorouacil (5-FU), doxorubicin) investigated various combinatorial regimens efficacy against colorectal cancer cells [13]. The authors demonstrated that PDTC and vitamin E decrease tumorigenicity in vitro and in vivo by sensitizing cells to 5-FU and doxorubicin through the induction of a G_1_ cell-cycle arrest, and subsequent induction of cell death. Similar findings using compounds with antioxidant capacity and cytotoxic chemotherapies (e.g., cisplatin) were observed in cervical, ovarian, and lung cancer cells as well [14]. However, despite these promising findings suggesting synergy between chemotherapeutics and antioxidants against tumor cells, more recent findings depict numerous molecular mechanisms demonstrating that alterations in the intracellular antioxidant machinery in tumor cells facilitate cancer cell survival and promote resistance to chemotherapeutic agents [15].

**Figure 1:**
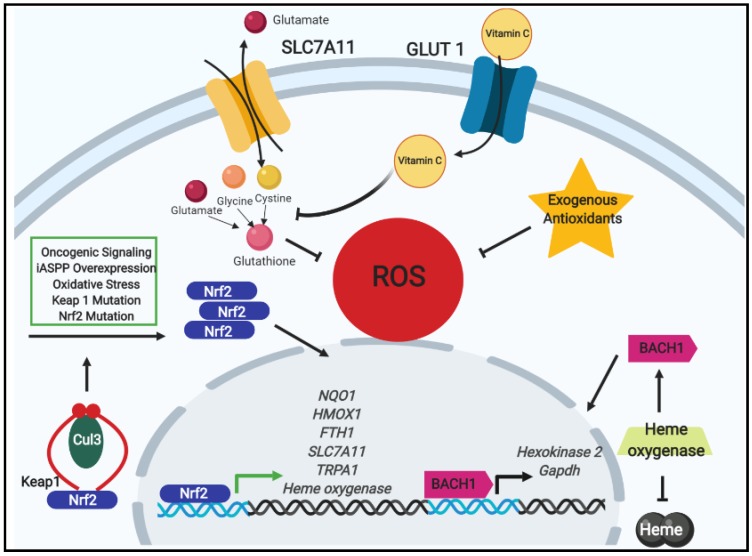
The impacts on tumor progression by oxidative stress regulation by nuclear factor erythroid 2-related factor 2 (Nrf2) signaling and exogenous antioxidants. Under normal conditions, Nrf2 is bound in a complex with kelch-like ECH associated protein 1 (Keap1) (red curves) and cullin-3 (Cul-3) containing E3 ubiquitin ligase (green oval) where it is targeted for proteasomal degradation. Nonetheless, upon increases in stimuli that promote Nrf2 stabilization (e.g., oxidative stress, oncogenic signaling, mutations in Nrf2 or Keap1 prohibiting their binding, or interference of the Nrf2–Keap1 interaction by iASPP), Nrf2 accumulates in the cytosol and translocates to the nucleus wherein it binds to the antioxidant response element (ARE) and initiates an antioxidant program. Among the genes transcribed by Nrf2 is the cystine/glutamate antiporter *SCL7A11*. With its upregulation due to Nrf2, extracellular cystine uptake increases, and when combined with glycine (orange sphere) and glutamate (red sphere), the antioxidant glutathione is synthesized and contributes to decreasing reactive oxygen species (ROS) leading to tumor progression and drug resistance. Exogenous antioxidants likewise promote tumor progression by decreasing intracellular ROS levels. When oxidative stress is reduced, Nrf2 promotes the transcription of heme oxygenase which catabolizes free heme and stabilizes basic leucine zipper transcription factor 1 (BACH1). Thereafter, BACH1 induces glycolysis-dependent metastasis via the transcription of *Hexokinase 2* and *Gapdh*. Lastly, in the case of the antioxidant vitamin C, which enters cells via the glucose transporter GLUT1, diminishing glutathione levels were observed to occur leading to subsequent increase in energetic crisis and cell death.

For instance, in a number of distinct cancer contexts, the stabilization of the nuclear factor erythroid 2-related factor 2 (Nrf2) transcription factor, a master regulator of the intracellular antioxidant program and redox homeostasis, can facilitate the development of chemoresistance [16]. Under normal conditions, Nrf2 activity is tightly restricted by binding with kelch-like ECH associated protein 1 (Keap1). Keap1 functions as an adaptor protein to promote the interaction of cullin-3 (Cul-3)-based CRLs (cullin RING ligases) with Nrf2 in fashion that leads to ubiquitination and proteasomal degradation (Figure 1). Upon exposure to oxidative stress, the interaction between Nrf2 and Keap1 is disrupted and the Nrf2 transcription factor accumulates in abundance and translocates to the nucleus. Nrf2 can bind to genes containing antioxidant response elements (AREs) which encode a multitude of antioxidative and cellular defense targets. These include NADPH quinone oxidoreductase 1 (NQO1) [17], heme oxygenase-1 (HMOX1) [18], ferritin heavy polypeptide 1 (FTH1) [19], and the cystine/glutamate antiporter SCL7A11 [20], Additionally, in efforts to reduce ROS accumulation that result from processes such as proliferation and metabolism, signals emanating from the activated oncogenes Kras, Braf and Myc were identified to stabilize Nrf2, induce the antioxidant program, and subsequently decrease intracellular ROS [21].

More recently, a number of elegant studies have revealed that alterations in cancer cells that promote the stabilization of Nrf2 and a concomitant antioxidant response can promote chemotherapeutic resistance. Interestingly, upregulation of iASPP, a known inhibitor of p53, is correlated with the advanced stages of multiple malignancies, such as glioma, prostate cancer, and melanoma [22]. It also has been demonstrated to confer chemotherapeutic drug resistance in breast, hepatocellular, and ovarian malignancies [23]. However, evidence that iASPP has a cytosolic function (independent of p53 binding) led investigators to examine mechanisms by which iASPP could promote tumor progression and chemoresistance while localized in the cytosol. Interestingly, iASPP was found to lower the levels of ROS in cancer cells by competing with Nrf2 for Keap1 binding. This competition ultimately leads to a diminished interaction between Nrf2 and Keap1 which prevents ubiquitination and promotes Nrf2 accumulation. Nrf2 transcription can then coordinate an antioxidant response that promotes cancer cell growth as well as propels resistance to chemotherapeutics such as 5-flurouracil (5-FU) [24]. To substantiate the relationship between resistance to 5-FU and a Nrf2-mediated antioxidant response, the investigators first demonstrated that siRNA-mediated knockdown of iASPP led to an increase in 5-FU-mediated apoptosis. The iASPP knockdown-mediated increase in apoptosis is completely ameliorated through exogenous addition of N-acetylcysteine (NAC), allowing the conclusion that 5-FU-mediated apoptosis in iASPP-deficient cells is dependent on ROS production.

In further support of the idea that antioxidant capabilities can be beneficial for cancer cells are multiple, recent, high profile studies that link antioxidant activity and tumor progression. This area of inquiry was perhaps initiated by a seminal study published in 2011 demonstrating a direct relationship between activation of K-Ras, B-Raf, or Myc, an increase in Nrf2 levels, and the consequent elimination of ROS [21]. This study also demonstrated that genetic ablation of Nrf2 could impair the formation of pancreatic intraepithelial neoplasias (PanINs) in a genetically engineered mouse model (GEMM) of K-Ras-driven pancreatic cancer. BRCA1 was also demonstrated to be a Nrf2 interacting protein where it can a promote its stabilization and activation by interfering with KEAP1-mediated degradation [25]. When BRCA1 is lost, estrogen receptor (ER) signaling can promote Nrf2 stability. However, ER-negative breast cancers with BRCA1 deficiencies could thus be vulnerable to agents that promote oxidative stress. More recently, Nrf2 activation was found to be a consequence of treatment with anti-diabetic drugs [26]. As a result, Nrf2 can promote metastasis (without impacting the incidence of primary tumors) in immunocompromised mice. Taken together, these studies suggest that multiple mechanisms may converge on Nrf2 to promote tumor progression and metastasis in different contexts.

### 2.2. Exogenous Antioxidants and Oncogenic Signaling in Tumor Progression

Additionally, recent research has demonstrated that high doses of vitamin C induce cell death in colorectal cancer cells with oncogenic mutations in K-Ras or B-Raf [27]. The mechanism of death induced by vitamin C in these cells involves the elevation of ROS level. The authors convincingly demonstrate that vitamin C is oxidized in the extracellular space to dehydroascorbate (DHA) where it can then enter cancer cells through the GLUT1 receptor. As a consequence of this DHA uptake, the cell mounts a vigorous antioxidant response to neutralize DHA back to Vitamin C, and thus there is a consequent depletion of the intracellular antioxidant glutathione (Figure 1). Interestingly, the elevated ROS levels result in an inactivation of glyceraldehyde-3-phosphate dehydrogenase (GAPDH) which ultimately leads to an energetic crisis that kills these highly glycolytic cancer cells. The mechanism underlying the capacity of vitamin C to kill cancer cells seems to be somewhat of a paradox on the surface given the well-established antioxidant capabilities of vitamin C itself [28]. That being said, these findings are entirely consistent with long-standing, authoritative studies demonstrating both pro- and antioxidant capabilities for vitamin C in distinct contexts [29,30].

Expanding on these exciting findings, multiple studies have now contrasted the impact of antioxidant activity on cancer cells present in metastatic lesions with the necessity of ROS neutralization at the site of the primary tumor. In K-Ras and B-Raf-driven mouse models of lung cancer, it was discovered that dietary antioxidant supplementation could enhance tumor progression and reduce survival [31]. Mechanistically, the authors discovered that antioxidant activity could reduce p53 accumulation and thereby stimulate tumor progression. In addition to driving the early development of tumors, exogenous antioxidants have also been observed to promote metastasis. In two recent studies of lung cancer cells, the pro-metastatic basic leucine zipper transcription factor 1 (BACH1) has been found to promote metastasis in KRAS-driven tumor cells. Free intracellular heme induces BACH1 degradation by promoting its targeting to the ubiquitin ligase Fbxo22. From one study, Nrf2 was found to stabilize BACH1 by inducing heme oxygenase 1 (Ho1) activity [32]. Ho1 promotes the catabolism of free intracellular heme and leads to full length BACH1 that is found in the nucleus and aids in inducing pro-metastatic genes. The second study complemented this finding by investigating how BACH1 stabilization leads to metastasis under reduced oxidative stress levels [33]. In their discovery, investigators found that long term exposure to exogenous antioxidants leads to lower levels of oxidative stress and free heme. This induces BACH1-driven transcription of *Hexokinase 2* (HK2) and *Gapdh* which results in metastasis dependent upon glucose metabolism (Figure 1). On the same note of antioxidants promoting metastasis, two papers were published examining the relationship between antioxidant activity and melanoma metastasis [34,35]. Both of these studies found compelling evidence that diminishing oxidative stress could facilitate tumor cell migration and invasion. In one case, the authors observed a profound increase in migration and invasion that was dependent on new glutathione synthesis and increased activation of the small guanosine triphosphatase RHOA [34]. This enhanced invasion resulted in a significant increase in lymph node metastases in mice harboring conditional activation of B-Raf and conditional deletion of PTEN. Similarly, using patient-derived xenografts in NOD/SCID IL2Rγ^null^ (NSG) mice, the other study demonstrated that NAC treatment promoted melanoma metastasis while having no significant effect on the size of the primary, subcutaneous tumors [35]. The underlying mechanism in this study was slightly distinct from the previous study, likely owing to the vastly distinct models that were used in each instance. In this case, metastasizing melanoma cells were found to rewire metabolism in a fashion that rendered these cells highly dependent on NADPH generated from enzymes in the folate pathway, to successfully metastasize. Taken together, these studies reveal oxidative stress to be an obstacle to tumor progression and metastasis in both lung cancer and malignant melanoma and suggest that exogenous antioxidant supplementation can aid tumor cells in navigating the metastatic cascade.

## 3. Antioxidant Activity, ECM-Detachment, and Rewired Metabolic Circuitry

### 3.1. Metabolic Influence and ROS Reduction in ECM-Detached Tumor Cells

As discussed above, there is now abundant evidence indicating that antioxidant activity can promote tumor progression and metastasis in certain contexts. While there are undoubtedly numerous mechanisms underlying this relationship between diminished ROS and tumor progression, one context in which antioxidant activity has been found to be particularly important is in the absence of integrin-mediated attachment to extracellular matrix (ECM). Normally, ECM-detachment results in the activation of a caspase-dependent cell death process known as anoikis [36]. However, as discussed in more detail below, the inhibition of anoikis is not sufficient to promote the long-term viability of ECM-detached cells [5,37]. Indeed, the viability of ECM-detached cells is intimately dependent on the rewiring of cellular metabolism to promote energy generation and to diminish ROS. These findings have significant implications for tumorigenesis as cancer cells will be exposed to ECM-detached conditions at multiple points during the course of tumor progression [38]. Indeed, there is now a plethora of evidence linking oncogenic signaling to both inhibition of anoikis and metabolic rewiring elicited by detachment from extracellular matrix [39,40].

The observation that ECM-detached cells lacking the capacity to undergo anoikis could still die in a programmed fashion was made in MCF-10A cells grown in three-dimensional culture as mammary acini [37,41]. Studies aimed at understanding this non-apoptotic death revealed that ECM-detachment is also a signal for significant changes in metabolism [5]. More specifically, ECM-detachment results in a decrease in both glucose and glutamine uptake [42], shuttling flux through the pentose phosphate pathway (PPP), promoting NADPH production, and consequent reduction of ECM detachment-induced ROS. Intriguingly, neutralization of ROS through exogenous supplementation of antioxidants is sufficient to restore ATP levels and promote survival of ECM-detached cells regardless of changes in glucose uptake. These findings raise the possibility that the activity of endogenous antioxidant defenses could be disproportionately important during ECM-detachment. Follow up studies reveal that this is indeed the case as shRNA-mediated reduction in the levels antioxidant enzymes (catalase and superoxide dismutase) facilitates the survival of breast cancer cells during ECM-detachment [43]. Moreover, overexpression of antioxidant enzymes promoted the survival of ECM-detached cells in the luminal space of mammary acini. Conversely, silencing antioxidant enzyme expression in breast cancer cell lines resulted in deficient anchorage-independent growth. Additional experiments utilizing tail vein injections in immunocompromised mice demonstrated that cancer cells deficient in antioxidant enzymes had a diminished capacity to develop tumors.

**Figure 2:**
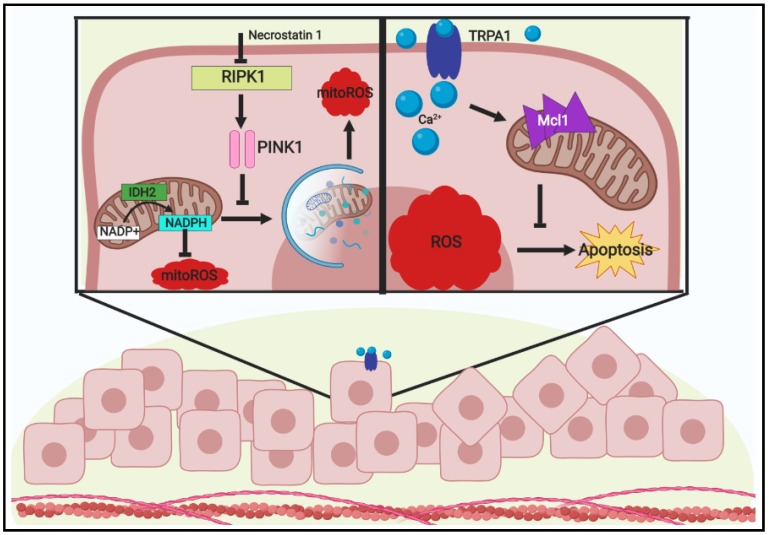
Mitigation and tolerance of ROS within extracellular matrix (ECM)-detached cancer cells Detachment from the extracellular matrix is a known stimulus for oxidative stress accumulation. Since continued ROS accumulation is understood to induce apoptosis in cells, cancer cells must employ mechanisms to address this obstacle and proceed in growth. Among these mechanisms lies two modes of addressing ROS during ECM-detachment: mitigation or tolerance. Through investigations of ECM-detachment-induced cell death, receptor-interacting protein kinase 1 (RIPK1) was surprisingly found to regulate mitophagy by promoting PINK1 stabilization and resulting in an increase in mitochondrial ROS (mitoROS). Upon inhibition or absence of RIPK1, PINK1 is susceptible to cleavage and mitoROS decreases through the reducing effects of IDH2 to generate NADPH. Additionally, increases in the Ca^2+^ transporter TRPA1 have been observed in a variety of cancers. During ECM-detachment, TRPA1 was observed to promote the increase in Ca^2+^ influx into cells which lead to increases in the levels of antiapoptotic Mcl1 and ultimately resulted in evasion of apoptosis despite the presence of high ROS levels. Thus, tumor cells are enabled to be tolerant of oxidative stress during ECM-detachment by the aid of TRPA1.

Over the years, additional studies have uncovered additional mechanisms involved in redox homeostasis during ECM-detachment. In addition to the observed changes in glucose metabolism, glutamine oxidation was found to be impaired in ECM-detached cells. Instead, reductive formation of glutamine-derived citrate is enhanced in a fashion that is highly dependent on cytosolic isocitrate dehydrogenase-1 (IDH1) [44]. In addition, inhibition of IDH1 promoted mitochondrial ROS and reduced anchorage-independent growth. Furthermore, isotope tracing analysis revealed that reductive generation of isocitrate/citrate in the cytosol enters the mitochondria where it can be oxidized by IDH2. As a consequence, NADPH is produced in the mitochondria which assists in neutralizing mitochondrial ROS and enhancing anchorage-independent growth. In addition to the capacity of reductive carboxylation to mitigate ROS during ECM-detachment, recent work has found that receptor-interacting protein kinase 1 (RIPK1) signaling is intimately involved in redox signaling during ECM-detachment [45]. While it is well known for its capacity to regulate necroptosis [46], RIPK1 during ECM detachment results in enhanced stability of PINK1 protein and the downstream induction of mitophagy (Figure 2). Interestingly, mitophagy during ECM-detachment causes an increase in the production of mitochondrial ROS. While the precise mechanism by which this occurs is not yet clearly articulated, the elimination of mitochondria leads to an aggregate loss of IDH2-mediated NADPH production, which contributes to higher levels of ROS. In addition, cells engineered to be deficient in RIPK1-mediated mitophagy are better able to form tumors after tail vein injection into immunocompromised mice, suggesting that the capacity of RIPK1-mediated mitophagy to produce ROS may function in a tumor suppressive capacity.

### 3.2. ECM-Detachment and ROS Tolerance

Despite the fact that all of the aforementioned studies in ECM-detached cells involve altered intracellular metabolism to regulate the antioxidant machinery, recent work has uncovered a mechanism that permits tolerance to oxidative stress in the absence of alterations in the abundance of ROS (Figure 2). Focusing on ECM-detached cells found in the lumen of breast and lung spheroids (where there is evidence of higher levels of ROS production), the investigators discovered a novel Nrf2 target involved in ROS tolerance. While the signal to activate Nrf2 is indeed the elevation of ROS, the transcriptional target is not an antioxidant enzyme, but rather the cation channel TRPA1 [47]. TRPA1 promotes an increase in Ca^2+^ influx in response to ROS generation which results in elevated expression of the anti-apoptotic protein Mcl-1 and cancer cell survival. While TRPA1 is a direct transcriptional target of Nrf2 and does respond to the elevation of ROS levels, this mechanism by which higher levels of ROS are tolerated does not appear to involve regulation of the quantity of ROS. Instead, this mechanism counteracts the capacity of ROS to trigger apoptosis by inducing upregulation of Mcl-1. Extending these findings to sensitivity to cancer therapies, TRPA1 was found to promote resistance to the traditional, platinum-based chemotherapeutic carboplatin through a mechanism dependent on the Ca^2+^-dependent anti-apoptotic mechanism. This observation aligns with other studies investigating the relationship between chemotherapeutic treatments and antioxidant activity and provides additional evidence of circumstances in which antioxidant response programs can protect cancer cells from death induced by chemotherapy.

### 3.3. Metabolic Reprogramming and Alleviation of Oxidative Stress

**Figure 3:**
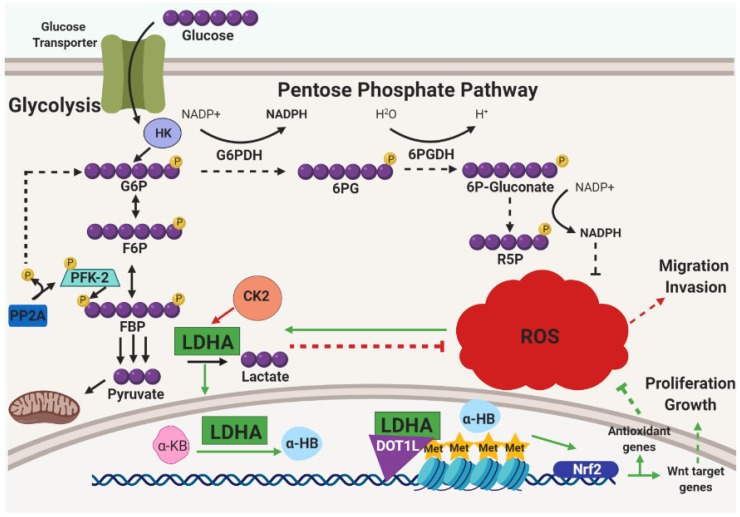
Glucose metabolism is highly crucial for cancer cells. This six-carbon monosaccharide can not only be used to fuel the energetic needs of cancer cells, but also to address the oxidative stress that accumulates as a byproduct of proliferation, metabolism, and growth. One mechanism employed to address oxidative stress is the use of the serine/threonine-protein phosphatase 2A (PP2A). During glycolysis, 6-phosphofructo-2-kinase (PFK-2) drives the commitment of glucose flux through the glycolytic pathway by influencing the phosphorylation of fructose-6-phosphate (F6P) and converting it into fructose-1,6,-bisphosphate (FBP). PP2A can reverse the flux of glucose to flow into the pentose phosphate pathway (PPP) by de-phosphorylating PFK-2. When this occurs, an increase of NADPH is generated and subsequent decreases in ROS is observed. The mechanism in this pathway is denoted by solid, black arrows, while the consequences of de-phosphorylated PFK-2 are marked by dashed, black arrows. Along the same note of rewired metabolism to address ROS accumulation, constitutive activation of casein kinase 2 (CK2) has been observed in many cancers, yet its mechanism was poorly understood. Recently, it was found that CK2 activity is dependent upon glucose metabolism, and from glycolysis, CK2 signaling relies on the metabolic enzyme lactase dehydrogenase (LDHA) to promote cancer cell migration and invasion. The CK2/LDHA signaling pathway is marked by solid, red arrows and the results of this signaling is marked by dashed, red arrows. Intriguingly, although LDHA promotes the conversion of pyruvate into lactate at the end of glycolysis in the cytosol, oxidative stress induces its translocation to the nucleus wherein it promotes the conversion of α-ketobutyrate (α-KB) into α-hydroxybutyrate (α-HB) which promotes a complex formation with LDHA and disruptor of telomeric silencing 1-like (DOTL1) to drive a Nrf2-dependent transcription of antioxidant genes as well as Wnt target genes. The consequence of these upregulated genes is diminished ROS levels and promotion of tumor growth. The ROS-induced LDHA translocation pathway is marked by solid, green arrows, while the consequences are indicated with dashed, green arrows. Other Abbreviations: glucose-6-phosphate (G6P); hexokinase (HK); glucose-6-phosphate dehydrogenase (G6PDH); 6-phosphogluconolactone (6PG); 6-phosphogluconate (6P-Gluconate); ribose-5-phosphate (R5P).

Furthermore, metabolic reprograming is not limited to ECM-detachment and is, more broadly, considered an emerging hallmark of cancer cells used to promote cell growth, survival, proliferation, and maintenance [48]. Metabolic reprogramming also renders cancer cells dependent on specific metabolic enzymes or pathways which creates possible vulnerabilities that could be exploited in cancer therapy [49]. Generally, tumor cells increase their uptake of glucose and upregulate their use of glycolysis even when in the presence of oxygen with functioning mitochondria, a phenomenon commonly known as the Warburg effect [50]. In addition, adaptation to metabolic alterations in tumors not only includes aerobic glycolysis, but also entails balancing needs for biosynthesis (e.g., nucleotide synthesis) and redox homeostasis [48]. A number of elegant studies have unveiled important new mechanisms regarding how cancer cells achieve this balance. For example, in the case of malignant B cells, a key component is the serine/threonine-protein phosphatase 2A (PP2A) [51], which is known to function as a tumor suppressor in a number of distinct cancers [52]. Surprisingly, this study demonstrated an essential role for PP2A activity in B cell malignancies. When examining the mechanism underlying this dependency on PP2A, the investigators discovered that PP2A is necessary to direct glucose-derived carbon into the PPP in order to generate NADPH and combat oxidative stress. Investigators found PP2A to de-phosphorylate the metabolic enzyme 6-phosphofructo-2-kinase (PFK-2) and influence the conversion of fructose-1,6-bisphophate (FBP) into fructose-6-phosphate (F6P) which then fluxes glucose to the PPP (Figure 3). Interestingly, PPP flux is constitutively low in B cells due to the activity of the transcription factors PAX5 and IKZF1. These factors are known to transcriptionally repress G6PDH and other key PPP enzymes which consequently causes low levels of PPP flux, a reduction in NADPH production, and an elevation in ROS. Thus, B cell malignancies may harbor a unique vulnerability to agents that manipulate redox homeostasis that is ultimately controlled by the activity of PP2A.

Other recent studies have observed additional non-traditional mechanisms by which metabolic pathways are altered in cancer cells in a fashion that promotes redox homeostasis. For example, inhibition of lactase dehydrogenase A (LDHA), canonically known for its role in converting pyruvate to lactate at the final steps of glycolysis, was found to cause an elevation in intracellular ROS that are derived from oxidative phosphorylation [53]. More recently, LDHA inhibition was investigated further alongside the overexpression of casein kinase 2 (CK2), a serine/threonine kinase involved in a large number of cellular processes related to cell cycle activity and apoptosis [54]. Upon CK2 overexpression, investigators found a decrease in cell survival and anchorage-independent growth that could be partially rescued by treatment with the antioxidant NAC [55]. CK2 activity was found to be contingent upon LDHA activity which mitigated ROS and enhanced migration and invasion (Figure 3). In a similar vein, LDHA has also been surprisingly discovered to translocate to the nucleus in HPV16-positive cervical tumors as a consequence of HPV-E7-mediated ROS accumulation [56]. Unexpectedly, nuclear LDHA acquires non-canonical enzymatic activity to produce α-hydroxybutyrate (α-HB) from α-ketobutyrate (α-KB) and forms a complex with DOT1L (disruptor of telomeric silencing 1-like) that triggers histone H3K79 hypermethylation (Figure 3). Antioxidant genes were observed to be upregulated by this complex that was dependent on Nrf2 which also transcribed Wnt target genes to promote proliferation and cell growth. Thus, there are multiple mechanisms by which LDHA is involved in redox homeostasis in distinct cancers. Taken together, these data raise the possibility that LDHA could be a target for agents designed to limit tumorigenesis by interfering with antioxidant defenses.

### 3.4. Intracellular Deficiencies in Glutathione Metabolism

Another gene known to functionally link alterations in metabolism in cancer cells to a vulnerability to alterations in redox metabolism is ARID1A [57]. Loss of function mutations in ARID1A, a component of the SWI/SNF chromatin-remodeling complex, are common in multiple types of ovarian carcinomas. Interestingly, as a consequence of deficient ARID1A protein, the SLC7A11 cystine/glutamate antiporter is not transcribed, and these cancer cells consequently lack robust levels of intracellular cystine. Cysteine is required for synthesis of the antioxidant glutathione (GSH) [58,59], and import of dimeric cystine from the extracellular milieu through the SLC7A11 can significantly impact GSH levels. Thus, cancer cells deficient in ARID1A are sensitive to ROS-mediated death caused by agents that antagonize the (already diminished) levels of GSH. Notably, the size of xenograft tumors cause by injection of ARID1A-deficient cells is substantially reduced upon treatment with inhibitors of GSH. ROS production increases, cell growth diminishes, and apoptosis is induced.

Multiple additional studies also touch on glutathione synthesis as a potential vulnerability in cancer cells. GSH synthesis was initially identified as critical for tumor initiation where interfering with GSH production ahead of tumor initiation could slow tumor growth [60]. This vulnerability was lost at later stages of tumorigenesis, presumably due to activation of compensatory mechanisms in invasive cancer cells. Remarkably, strategies designed to inhibit both GSH and the thioredoxin pathway were successful at hindering cancer cells in both in vitro and in vivo experiments. These findings suggest that targeting both GSH synthesis and compensatory antioxidant signaling may be efficacious in impeding tumor growth. Elegant studies in breast cancer cells have revealed that activated PI(3)K signaling can promote stabilization of Nrf2 and lead to enhanced levels of GSH [61]. Interestingly, tumors derived from breast cancer cells with hyperactivating mutations in PI(3)K regress following dual treatment with GSH synthesis antagonists and cisplatin. While the aforementioned study focused on breast cancer cells, it is clear that cancer cells derived from a number of distinct sources have differing sensitivities to GSH inhibition. In an effort to understand the factors that determine the sensitivity of cancer cells to GSH inhibition, recent work demonstrated a significant role for deubiquitinating enzymes (DUBs) [62]. Inhibition of DUB function causes an accumulation of ubiquitinated proteins, an increase in proteotoxic stress, and sensitizes a number of cancer cell lines to death induced by GSH inhibition.

### 3.5. Iron Accumulation and ROS

Similar to the vulnerability to GSH inhibitors discovered in ARID1A-deficient cancer cells, the iron exporter ferroportin (FPN) has been observed to be dysregulated in tumor cells. Later stage breast, prostate, ovarian, colorectal, and multiple myeloma cancers have been associated with low FPN expression relative to neighboring healthy tissues [63,64,65,66,67,68]. Iron metabolism is of particular interest in understanding potential vulnerabilities of cancer cells to agents that alter the redox balance given that the presence of ferrous iron in the cytosol can be a significant contributor to ROS generation produced by the Fenton reaction [69]. Thus, diminished levels of ferroportin could function as a de facto antioxidant for cancer cells in which the limited intracellular iron pool results in a deficiency in Fenton-mediated production of ROS. A recent study has attempted to exploit this potential vulnerability using an FDA-approved iron oxide nanoparticle, ferumoxytol, currently used to treat iron deficient conditions [70]. Using leukemia cells that express low levels of ferroportin, the investigators discovered that these cells are particularly susceptible to death caused by ferumoxytol treatment. Importantly, cell death caused by ferumoxytol treatment was demonstrated to be a consequence of an elevation in intracellular iron and a concomitant increase in both cytosolic and mitochondrial ROS accumulation. Extending these findings to pre-clinical experiments, ferumoxytol treatment caused a significant reduction of tumor burden in a murine leukemia model as well as patient-derived xenograft transplants bearing leukemia cells with low ferroportin expression. These findings represent another instance of how the reliance of various cancer cells on antioxidant defenses can be exploited for therapeutic gain. Of particular interest is that this nanoparticle, which was once considered biologically inert, could be utilized in clinical trials for patients with leukemias characterized with low ferroportin levels. Additionally, iron is linked to cell death caused by ferroptosis, a recently described form of programmed cell death that is functionally and morphologically distinct from apoptosis and necroptosis [71]. Given that this type of cell death also involves generation of ROS and that there are documented instances of ferroptosis resistance in cancer cells [72], it stands to reason that antioxidant activity in cancer cells could promote survival by blocking ferroptosis.

## 4. Dietary Prooxidants

### Diet-Derived Prooxidants and Tumor Progression

One of the critiques of the unsuccessful clinical trials examining the effects of antioxidants on individuals at high risk for cancer was the use of pharmaceutical reagents as opposed to antioxidants derived from normal dietary components. Many natural compounds are a genuine source of bioactive compounds and many have been demonstrated to have anti-cancer activity [73,74]. With regards to antioxidant activity and in light of the studies that have been discussed above, one could surmise that a diet-derived compound that could be efficacious for cancer treatment would target cancer cells by increasing levels of intracellular ROS and thereby decreasing the capacity for cancer cells to survive. One potential class of compounds with these traits are rosemary polyphenols that have displayed a vast antiproliferative capacity against colon cancer cells in vitro and in animal models [75,76]. Intriguingly, colorectal cancer cells treated with rosemary extract (RE) displayed diminished proliferation, migration, colony formation, and enhanced necrotic cell death due to an acute elevation in the levels of intracellular ROS. Moreover, when used in xenograft models of colon cancer, a significant reduction in tumor growth was observed in mice that received oral supplementation of RE. These data suggest that RE treatment may be a particularly interesting strategy to exploit the significant reliance of certain cancer cells on endogenous antioxidant programs. However, additional efforts aimed at understanding the molecular mechanisms by which RE treatment can cause an elevation in ROS are necessary.

In addition to RE, the naturally occurring compound phenethyl isothiocyanate (PEITC), found mostly in cruciferous vegetables, has been investigated for anti-cancer activity by modulating intracellular ROS [77]. In transformed cancer cells, it was found that PEITC conjugates with endogenous GSH, causes a depletion of GSH levels and subsequent oxidative stress and cytotoxicity result from the treatment. Furthermore, PEITC has been registered in clinical trials for cancer prevention and treatment indicating that PEITC could possibly hold a therapeutic promise to treat cancer patients. In a recent study [78], a series of small, molecular analogs of PEITC were synthesized to discover a more potent ROS modulator with improved anticancer capacity. Cancer stem-like cells (CSCs) are believed to be critical (in some cancers) for tumor initiation, development, metastasis and/or recurrence, and previous studies have demonstrated that elevated levels of ROS may be effective at inducing cell death in CSCs [79]. The investigators used one of their newly synthesized compounds, LBL21, and found a significant decrease in the levels of intracellular GSH, which subsequently leads to ROS accumulation and mitochondrial dysfunction. Notably, the activity of LBL21 was quite effective at eliminating the subpopulation of non-small cell lung cancer A549 cells. Further functional studies revealed that LBL21 inhibited the ability of cancer cells to form colonies in vitro and develop tumors in vivo. Ultimately, the data suggest that LBL21 possesses promising anti-cancer activity due to specificity in eliminating CSCs. Other compounds from cruciferous vegetables with documented anti-cancer activity include indole-3-carbinol (I3C) which recently was found to reactivate PTEN by inhibiting the function of the WWP1 ubiquitin ligase [80]. While this study did not investigate a relationship between I3C and redox metabolism, other studies in opportunistic human pathogens have revealed that I3C can function to promote an increase in ROS levels [81]. Thus, future studies aimed at understanding if I3C can cause elevated ROS levels in cancer cells may be fruitful in the discovery of natural compounds that can exploit the vulnerability of certain types of cancer cells to manipulation of redox metabolism.

## 5. Conclusions

As discussed here, oxidative stress can function as a barrier to tumor progression due to its capacity to induce deleterious changes to cancer cells. Although antioxidant supplementation was once thought to provide clinical benefits for cancer patients, the data to support such a model are decidedly mixed. Mounting evidence suggests that antioxidant supplements can aid tumor cells much in the same way that they can aid normal cells. Additionally, rewiring of the endogenous antioxidant program can significantly impact the sensitivity of cancer cells to any exogenous manipulations in levels of oxidative stress. Among the most important factors is Nrf2, as its activity is observed to play a crucial role in promoting cancer cell survival and chemotherapeutic resistance in numerous and distinct contexts. This activity includes canonically inducing the transcription of antioxidant genes as well as promoting increased calcium uptake to inhibit apoptotic induction and tolerate intracellular ROS. In addition to promoting Nrf2 stabilization, oncogenic signaling can also engage factors responsible for migration, invasion, and metabolic reprogramming to produce reducing agents that neutralize ROS. Numerous perturbations in either intracellular antioxidant genes or genes that can influence redox homeostasis are present in cancer cells, and when targeted they reveal a vulnerability suggesting that low ROS are a requisite part of their survival. Taken together, the findings discussed here give credence to the idea that therapeutic approaches designed to elevate ROS levels may ultimately have some specificity towards tumor cells. Ultimately, despite the significant advances discussed here, much work remains to determine the precise context(s) are dependent on antioxidant activity. As things currently stand, the basic biology is not sufficiently understood to allow for meaningful translation of these findings into cancer patients. Nonetheless, the promise of selectively targeting the reliance of cancer cells on antioxidant activity for therapeutic gain is an exciting and innovative idea that warrants further investigation.
